# Oxidative Stress in Depression: The Link with the Stress Response, Neuroinflammation, Serotonin, Neurogenesis and Synaptic Plasticity

**DOI:** 10.3390/antiox12020470

**Published:** 2023-02-13

**Authors:** Ana Salomé Correia, Armando Cardoso, Nuno Vale

**Affiliations:** 1 OncoPharma Research Group, Center for Health Technology and Services Research (CINTESIS), Rua Doutor Plácido da Costa, 4200-450 Porto, Portugal; 2 Institute of Biomedical Sciences Abel Salazar (ICBAS), University of Porto, Rua de Jorge Viterbo Ferreira, 228, 4050-313 Porto, Portugal; 3 CINTESIS@RISE, Faculty of Medicine, University of Porto, Alameda Professor Hernâni Monteiro, 4200-319 Porto, Portugal; 4 NeuroGen Research Group, Center for Health Technology and Services Research (CINTESIS), Rua Doutor Plácido da Costa, 4200-450 Porto, Portugal; 5 Unit of Anatomy, Department of Biomedicine, Faculty of Medicine, University of Porto, Alameda Professor Hernâni Monteiro, 4200-319 Porto, Portugal; 6 Department of Community Medicine, Information and Health Decision Sciences (MEDCIDS), Faculty of Medicine, University of Porto, Alameda Professor Hernâni Monteiro, 4200-319 Porto, Portugal

**Keywords:** oxidative stress, reactive oxygen species, depression, stress response, neuroinflammation, serotonin, neurogenesis, synaptic plasticity

## Abstract

Depression is a prevalent, complex, and highly debilitating disease. The full comprehension of this disease is still a global challenge. Indeed, relapse, recurrency, and therapeutic resistance are serious challenges in the fight against depression. Nevertheless, abnormal functioning of the stress response, inflammatory processes, neurotransmission, neurogenesis, and synaptic plasticity are known to underlie the pathophysiology of this mental disorder. The role of oxidative stress in disease and, particularly, in depression is widely recognized, being important for both its onset and development. Indeed, excessive generation of reactive oxygen species and lack of efficient antioxidant response trigger processes such as inflammation, neurodegeneration, and neuronal death. Keeping in mind the importance of a detailed study about cellular and molecular mechanisms that are present in depression, this review focuses on the link between oxidative stress and the stress response, neuroinflammation, serotonergic pathways, neurogenesis, and synaptic plasticity’s imbalances present in depression. The study of these mechanisms is important to lead to a new era of treatment and knowledge about this highly complex disease.

## 1. Introduction

The importance of mental health has grown in recent times. In fact, it is imperative to look at this problem as a real and increasingly prevalent issue. Depression is a very common mental illness worldwide, where it is estimated that 5% of adults suffer from depression [1]. This disease is characterized by several symptoms, such as apathy or a predominantly sad and negative mood. Despite the existence of several therapeutic modalities, namely psychotherapy and antidepressants, there are several problems associated with the therapy of this disease, such as the development of resistance and relapses after the end of therapy. Additionally, as it is an extremely complex disease in which several biological systems are involved, it is sometimes difficult to find the most efficient therapy for each patient [2].

Thus, in-depth study of this disease is crucial. In fact, there are several cellular and molecular mechanisms involved, such as an exacerbated stress response, the presence of high levels of neuroinflammation, an imbalance in the signaling mediated by neurotransmitters (with a focus on serotonin), and, also, problems at the level of neurogenesis and synaptic plasticity, largely mediated by brain-derived neurotrophic factor (BDNF) [3].

All these factors are intensified by the presence of high levels of oxidative stress, and they can even lead to increased levels of this type of stress [3,4]. In fact, oxidative stress is a phenomenon caused by a disturbance in the normal balance in the production of free radicals, being connected to several diseases, such as diabetes and depression [5]. Indeed, the role of oxidative stress in depression is recognized [6]. Excessive reactive oxygen species generation and lack of efficient antioxidant response trigger processes such as inflammation, neurodegeneration, tissue damage, and cell death [6].

Our review aims to focus on the link between oxidative stress and the stress response, neuroinflammation, serotonin, neurogenesis, and synaptic plasticity in the context of depression. The detailed study of these topics is important to lead to a new era of treatment and knowledge about this disease.

## 2. Oxidative Stress: An Overview

Oxidative stress is a biological mechanism caused by a disturbance in the normal balance between the production of free radicals (particularly reactive oxygen species) and antioxidant defenses, important to maintain the normal production of free radicals by detoxifying these reactive species, produced in several metabolic reactions that may be enhanced by the exposure to environmental stressors such as smoking or ultraviolet (UV) radiation [5].

The main reactive oxygen species (ROS), superoxide radicals (O_2_^•−)^, hydrogen peroxide (H_2_O_2_), hydroxyl radicals (^•^OH), and singlet oxygen (^1^O_2_), are generated through processes such as immunity, apoptosis, protein phosphorylation, and other cellular signaling processes. Indeed, in the human body, ROS are mainly produced in mitochondria, peroxisomes, and the endoplasmic reticulum, being continuously generated by enzymatic reactions that involve cyclooxygenases, NADPH and xanthine oxidases, and lipoxygenases and through the Fenton reaction [7]. At low quantities, ROS are important to the maintenance of homeostasis and cellular processes [5,8,9]. However, when the production of these species increases in response to several stimuli such as pollutants and drugs, negative effects occur in cellular structures and processes. Indeed, proteins, lipids, nucleic acids, enzymes, cellular division, and cellular metabolism are highly affected, being associated with the development and progression of several diseases, such as cancer [5,8,10]. Intense oxidant exposure affects unspecific targets, promoting an imbalance in the adaptive pathways, such as nuclear factor-κB (NF-κB) and Nrf-2, converging into pathological conditions [11].

**Figure 1:**
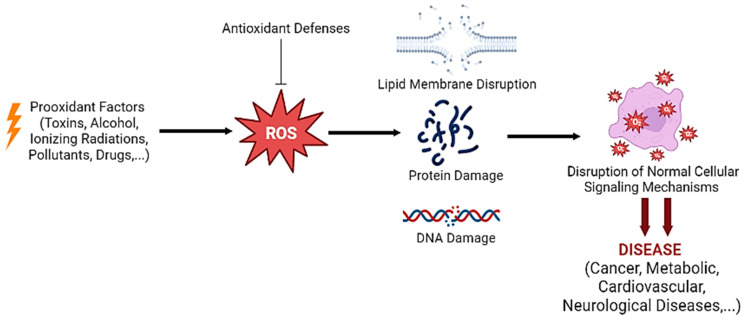
Prooxidant factors such as toxins and pollutants promote high levels of ROS production that are kept at low levels by the antioxidant defenses. However, when ROS production overwhelms antioxidant defenses, harmful effects such as membrane disruption, protein and DNA damage occur, disrupting the normal cell signaling mechanisms and culminating in diseases such as cancer or neurological diseases. Created with BioRender [14].

To protect from the harmful effects of high levels of ROS, cells have antioxidant defenses, particularly enzymatic systems such as superoxide dismutase (SOD), catalase (CAT), and glutathione peroxidase (GPx). In fact, endogenous and exogenous antioxidants play vital roles in the maintenance of a healthy organism, being important to maintain ROS homeostasis [5,12]. The production of ROS must be equalized by a similar rate of antioxidant consumption. However, in pathological conditions, ROS production overwhelms the antioxidant capability of the organism, leading to an imbalance that causes harmful effects in cells/tissues, promoting disease progression [8,13]. Figure 1 summarizes the harmful effects of excessive ROS production.

Several diseases such as cancer, diabetes, and cardiovascular and neurological pathologies originate/develop from the imbalance of the oxidative homeostasis. Indeed, high amounts of ROS promote cellular damage, culminating in pathological states (Figure 1). Thus, acute, chronic, or degenerative diseases may be speeded up or induced by uncontrolled levels of oxidative stress [5]. Understand the processes and role of oxidative stress in human diseases is important and urgent.

Epithelial–mesenchymal transition, genome instability, and metastasis are features associated with cancer and influenced by ROS production [7]. Indeed, overproduction of ROS is known to be crucial for carcinogenesis by promoting DNA oxidative damage, more specifically base alterations, abasic sites, and strand breaks [15]. Additionally, ROS promote the overactivation of cancer survival pathways, such as STAT-3, signal transducer and activator of transcription 3/VEGF, vascular endothelial growth factor, STAT-3/VEGF, signaling, that promotes angiogenesis [16]. Inflammation also promotes and is induced by ROS. For example, in a mouse model, TNF-α signaling induced by inflammatory responses induces the Noxo1, a component of the NADPH oxidase 1 (NOX1) complex, which produces reactive oxygen species (ROS), NOX1/ROS signaling pathway, that leads to the expression of tumor-promoting mechanisms, particularly stemness [17]. Pathways involved in cancer progression such as cyclin D1, extracellular signal-regulated kinase (ERK), JUN N-terminal kinase (JNK), and mitogen-activated protein kinase (MAPK) are also activated by ROS [18]. Lifestyle choices can also lead to oxidative stress, for example, by dietary fat consumption, enhancing lipid peroxidation, strongly correlated with cancer development [5]. Thus, cancer cells have aberrant redox homeostasis. Indeed, ROS induce carcinogenesis and are also cytotoxic at high levels [19,20].

Oxidative stress also plays an important role in the development of diabetes and its associated complications [21]. ROS can indeed deteriorate pancreatic islet β-cells, leading to impaired insulin secretion. Insulin resistance can also be promoted by several signaling pathways activated by ROS, such as NF-κB [22,23]. The constant hyperglycemia and high levels of ROS are also involved in the development of atherosclerosis, frequently observed as a diabetic complication [24]. Indeed, oxidative stress is highly connected to cardiovascular diseases such as atherosclerosis, acting as a trigger for this condition. Low-density lipoprotein (LDL) cholesterol is oxidized by ROS, leading to lipid accumulation by the formation of foam cells and ultimately to the formation of an atherosclerotic plaque [5]. These species can also promote matrix metalloproteinase activation, leading to plaque rupture [25]. Other cardiovascular diseases such as myocardial fibrosis, cardiac hypertrophy, heart failure, and myocardial infarction are also associated with increased ROS production [26].

Neurological disorders such as Parkinson’s disease (PD), Alzheimer’s disease (AD), Huntington’s disease, amyotrophic lateral sclerosis, and depression are also linked to oxidative stress [5,27]. For example, in AD, β-amyloid protein is formed by ROS action and is also a main source of these species, leading to neuronal loss, that is reflected in neurodegeneration [28]. Indeed, a study revealed that the methionine 35 residue is key to the oxidative stress and neurotoxic properties of this protein [28,29]. In PD, the loss of dopaminergic neurons is also connected with increased levels of oxidative stress. Indeed, the administration to animals of oxidative stress inducers such as rotenone and 6-hydroxydopamine (6-OHDA) increased dopaminergic neuronal degeneration [27,30,31].

Antioxidant defenses are important to counteract the harmful effects of oxidative stress. These can be enzymatic or nonenzymatic, as well as endogenous or exogenous. The latter are mainly introduced by the diet or nutritional supplementation [5]. Some examples of antioxidant defenses are vitamin C, vitamin E, carotenoids, catalase, and glutathione peroxidase [32]. Intense research on antioxidant-associated mechanisms and potentialities may lead to pharmacological success. Some examples in the context of depression will be described below.

## 3. Oxidative Stress and Depression

Depression is a globally prevalent disease, with an estimated 3.8% of the population affected. Indeed, a total of 280 million people in the world have this illness, being a worldwide concern [1]. This disease is characterized by several symptoms such as depressed mood, lack of interest or pleasure, fatigue, loss of energy, sleep disturbance, anxiety, and neurocognitive and sexual dysfunction, being highly debilitating. Ultimately, in severe cases, suicidal ideation may culminate in suicide [2].

This disease is very complex. Indeed, different systems are involved in the pathophysiological features of this disease, particularly the central nervous system, hypothalamic–pituitary–adrenal (HPA) axis, the autonomic nervous system, and the immune system [33,34]. The stress response, neuroinflammation, imbalance of neurotransmission, neurogenesis, and synaptic plasticity underlie the pathophysiology of depression [3]. Despite the well-known role of these features, the full comprehension of depression is still a major challenge [35]. Nevertheless, efficient therapy is available, particularly psychotherapy and antidepressant therapy. However, relapse, recurrency, and therapeutic resistance are serious challenges in the fight against this complex and debilitating disease [36].

**Figure 2:**
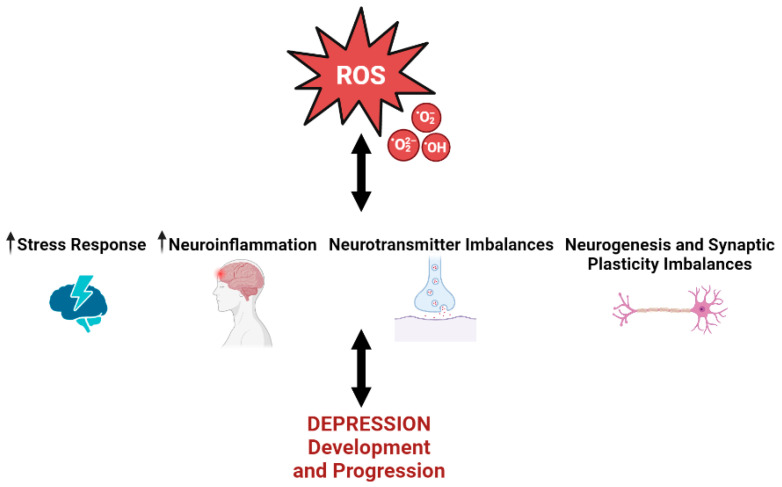
High levels of ROS promote and are promoted by the stress response, neuroinflammation, and imbalances in neurotransmitter-related pathways and neurogenesis/synaptic plasticity processes. All these features underlie the pathophysiology of depression, being critical in disease development and progression. Upward arrows represent an increment of the represented process. Created with BioRender [14].

The role of ROS in depression is well known. Studies reveal that depression is associated with lower intake of antioxidants such as vitamins A, C, and E, selenium and zinc, and B vitamins (B6, folate, and B12) [37]. Indeed, excessive ROS generation and lack of efficient antioxidant response trigger processes such as inflammation, neurodegeneration, tissue damage, and cell death [6]. Thus, oxidative stress is correlated with the pathogenesis and progression of depression. Evidence suggests that high levels of brain lipidic peroxidation and other parameters such as nitric oxide and ciclooxygenase-2 (COX-2) activity, important processes in the pathogenesis this disease, lead to high levels of oxidative stress. Together with the reduction of antioxidant defenses, these mechanisms highlight the role of oxidative stress as an important player in the development and progression of depression [4]. We will further focus on the high levels of ROS associated with the stress response, neuroinflammation, neurotransmitter imbalance, and neurogenesis/synaptic plasticity imbalance that underlie the development and progression of depression (Figure 2).

It is important to note that, being a highly complex and multifactorial disease, other processes are involved in depression’s pathogenesis. For example, hypoxia is involved in anxiety and depressive disorders, with few studies yet. Evidence points out that this process disrupts the neurohormonal homeostasis in the brain, increasing the potential for developing depression by promoting inflammation, apoptosis, dysregulation of serotonergic pathways, and also mitochondrial oxidative stress [38,39,40]. Targeting hypoxia-related pathways is a promising tool for chronic stress and depressive disorders [41]. Indeed, hypoxia contributes to high levels of oxidative stress [42], important in the context of depression, highlighted in this manuscript. In fact, we have recently found that hypoxia–ischemia induced an increase in ROS in neuron-like cells, and that the drug edaravone increased cell viability and reduced ROS production, probably by its free radical-scavenging properties [43].

### 3.1. Oxidative Stress and Depression’s Associated Stress Response

The HPA axis is the principal player in this response. This axis responds to physical or psychological stressors, releasing corticotrophin-releasing hormone (CRH) from the hypothalamus. This hormone, in turn, activates the pituitary gland to release adrenocorticotropin hormone (ACTH), that activates the adrenal gland to produce glucocorticoid and other players in the stress response, such as catecholamines [44]. In humans, the primary adrenal steroid is cortisol and in rodents it is corticosterone [45].

The connection between depression and the stress response is widely reported. Indeed, some studies report that some antidepressants downregulate the HPA axis, decreasing the degree of the stress response [46]. HPA axis dysfunction and elevated CRH mRNA expression levels are also reported in individuals with depression [47]. Prolonged exposure to high levels of glucocorticoids also results in synapse loss, neuronal death, and changes in neuronal dendrites [48]. High levels of stress were also reported to be connected with disruptions in serotonergic-related pathways, the volume of some brain areas such as the hippocampus and prefrontal cortex, and epigenetic changes in genes such as BDNF [49,50,51].

Increased production of ROS induces hyperactivation in the HPA axis [52]. Additionally, glucocorticoids released in response to HPA axis activation induce the activity of cellular reduction–oxidation systems. The activation of glucocorticoid receptors (GRs) in response to stress leads to an increase in mitochondrial membrane potential, calcium-holding capacity, and mitochondrial oxidation [53]. In turn, the production of superoxide, hydrogen peroxide, and hydroxyl radicals leads to oxidative stress, causing oxidative damage [54].

In vivo studies reported that after exposure to corticosterone, an increase in oxidative markers (such as lipidic peroxidation) and a decrease in antioxidant enzymatic systems (such as catalase) occurred. In this study, the oxidative injury in the hippocampus of rats led to impairment of their cognitive function [55]. Another study in mice also revealed that after the induction of depressive and anxiety-like behaviors with pressure injury, levels of corticosterone and brain oxidative stress markers were increased [56]. The administration of cholecalciferol (vitamin D3) to mice also counteracted depressive-like behavior and oxidative stress induced by repeated administration of corticosterone. Indeed, lipid peroxidation, protein carbonyl, and nitrite levels decreased after the treatment, improving depressive symptoms [57]. Another study in mice also supported the induction of high levels of oxidative stress markers by corticosterone, reversed by lutein, that exerted antidepressant-like effects in these animals [58]. The administration of the carotenoid crocin-I also alleviated neuroinflammation and oxidative damage induced by corticosterone, revealing an antidepressant activity. Indeed, this compound induced the activity of antioxidants, such as SOD-2 and glutathione reductase [59]. Catalpol administration to mice also inhibited HPA axis hyperactivity (reflected in lower levels of corticosterone, ACTH, and CRH), central inflammation, and oxidative damage via regulation of the NF-κB and Nrf2 pathways, producing antidepressant effects in mice injected with corticosterone [60]. The administration of *Myrcia pubipetala* Miq also exerted antioxidant effects in mice treated with corticosterone, that imbalanced the antioxidant enzyme activities in the hippocampus and cerebral cortex [61]. Another recent study evaluated the effects of day and night shift work on stress, anxiety, quality of life, and oxidative stress parameters in 60 nurses. Indeed, this study revealed that as the stress and anxiety levels increased, the amount of oxidant markers and cortisol levels increased, reducing the quality of life of the nurses [62]. The effect of L-cysteine on corticosterone-induced oxidative stress in rats was also antidepressant. L-cysteine reduced plasmatic corticosterone levels and increased antioxidant defenses, attenuating the oxidative stress promoted by corticosterone [63]. In vitro, the addition of *Hericium erinaceus* to rat pheochromocytoma cells (PC-12) also led to neuroprotective effects, relieving the oxidative stress caused by high doses of corticosterone. Indeed, this neuroprotective action was reflected by enhanced endogenous antioxidant enzyme activities, attenuated intracellular levels of ROS, and protection from apoptosis triggered by high levels of ROS [64].

All these studies support a clear connection between the overactivation of the HPA axis under stress conditions and oxidative stress in depression. Indeed, high levels of stress lead to decreased antioxidant levels and increased prooxidant levels, promoting the development and onset of depression. Table 1 summarizes data about the in vivo studies presented in this subsection.

### 3.2. Oxidative Stress and Depression’s Associated Neuroinflammation

Several studies have connected inflammation with depression. Indeed, elevated levels of immune markers such as granulocytes, monocytes, tumor necrosis factor (TNF), interleukin-6 (IL-6), and microglial activation are present in depressed individuals [65,66]. Another study revealed that the mRNA levels of proinflammatory markers (IL-1β, IL-6, TNF-α, and lymphotoxin A) and anti-inflammatory markers (cytokine IL-10, and of IL-1 receptor antagonist (IL-1RA)) were substantially increased and decreased, respectively, in the prefrontal cortex of depressed individuals who committed suicide [67]. Antidepressants such as imipramine also reduced microglial activation, decreasing proinflammatory cytokines levels, reversing stress-induced social avoidance in mice [68]. High levels of production of ROS in mitochondria are highly connected with inflammation, promoting oxidative stress, an important player in pathophysiology of depression. Indeed, several oxidative stress markers have been found to be increased in alcohol-induced aggressive and suicidal behaviors [69]. Thus, the immune system influences neuronal networks involved in depression, playing an important role in the pathogenesis of this disease.

Increased levels of oxidative stress generate dysregulation of the inflammatory response. Modifications of cell signaling promoted by oxidative stress lead to enhanced production of proinflammatory factors, promoting a proinflammatory response (particularly neuroinflammation, in the central nervous system), that is also modulated by oxidative stress [70]. By activating inflammatory pathways via nuclear factor-κB and mitogen-activated protein kinase family stress kinases, ROS are crucial in cell signaling. When present in excess, they promote cell damage and formation of proinflammatory molecules, such as malondialdehyde, ultimately leading to cell death. Indeed, an overactivated inflammatory system and increased levels of ROS act synergistically, promoting the onset and development of depression [71].

Several studies demonstrate this connection. Indeed, the administration to mice of the anti-inflammatory compound muscone ameliorated depression-like behavior by regulating inflammatory responses and improving oxidative stress markers, particularly malondialdehyde (MDA), SOD, and GPx [72]. In another study, the administration of apple phenolic extracts against lead acetate (Pb(Ac)2)-induced cognitive impairment and depression/anxiety-like behavior in mice revealed that the increased cellular oxidative damage and the levels of proinflammatory cytokines interleukin (IL)-1β, IL-6, and tumor necrosis factor-α were attenuated after the administration of apple phenolic extracts via the regulation of oxidative stress, neuroinflammation, and apoptosis via the miR-22-3p/Sirtuin 1 (SIRT1) signaling pathway [73]. Another similar study with cinnamic acid also improved lipopolysaccharide-induced depressive-like behaviors in mice by inhibiting neuroinflammation and oxidative stress. Indeed, proinflammatory cytokines (IL-6 and TNF-α) and oxidative stress markers (SOD, glutathione, and MDA) in the hippocampus and cortex of the depressed mice were highly improved due to the administration of cinnamic acid [74]. MDA is an agent that leads to protein damage and generation of advanced lipoxidation products with proinflammatory characteristics, detected in patients with depression [71,75,76]. Another recent study revealed that curcumin attenuated lipopolysaccharide-induced anxiety/depression-like behaviors in rats by decreasing cerebral oxidative stress. This compound increased the activity of SOD and GPx enzymes, as well as reduced MDA concentration. Additionally, it exerted anti-inflammatory properties by inhibiting microglial activation [77]. p-Coumaric acid is a compound that has a protective role against inflammation and oxidative stress (by scavenging ROS) in various diseases [78]. Indeed, another recent study revealed that this acid reverses depression-like behavior through inhibition of glycation end products (AGEs) and receptor for AGE (RAGE), AGE-RAGE-mediated neuroinflammation [79]. The administration of quercetin to zebrafish also alleviated oxidative stress and neuroinflammation induced by lipopolysaccharide (LPS), ameliorating behavioral abnormalities. Proinflammatory compounds such as TNF-α and IL-1β decreased and the antioxidant glutathione increased, versus LPS-treated fish [80]. Recently, targeting neuroinflammation using polyphenols was described as a promising therapeutic against inflammation-associated depression [81]. Indeed, polyphenols are plant-derived natural compounds with strong antioxidant properties [82]. In another study, these compounds were also shown to inhibit MAPK signaling pathway-mediated oxidative stress and inflammation in depression [83]. Similar results were obtained with LQFM212, 2,6-di-tert-butyl-4-((4-(2-hydroxyethyl)piperazin-1-yl)methyl)phenol, a piperazine derivative. This compound exhibited elevated antioxidant effects and also ameliorated LPS-induced behavioral, inflammatory, and oxidative changes in the tested animals [84]. Another study in humans aimed to investigate the levels of NOX1 and raftlin in depressed patients. NOX1 is an important source of ROS, and raftlin is important in inflammatory processes. The study concluded that depressed patients had increased serum NOX1 and raftlin levels compared to controls, highlighting the implication of oxidative stress and inflammatory processes in depression [85]. Resveratrol has also been linked to antioxidant, anti-inflammatory, and antidepressant effects. Indeed, a study revealed that this compound attenuated the depressive-like behavior in stressed animals, mediating alteration in the hippocampal levels of anti-inflammatory and proinflammatory cytokines and inducing antioxidant actions, such as the modulation of SOD and CAT activities [86]. In another study, the flower essential oil of Tagetes minuta also attenuated oxidative stress and restored the cellular pathway BDNF-Akt/ERK2, leading to an attenuation in inflammation and depressive-like behavior in mice [87]. The oxidative stress induced by indoor air pollution from solid fuel use also markedly increased inflammation, promoting depression and cognitive function impairment in middle-aged and older Chinese adults [88]. Another study also revealed that astilbin, that has antioxidant characteristics, ameliorated depressive-like behavior by regulating astrocyte-mediated neuroinflammation, caused by postnatal immune activation [89]. A study in humans also concluded that the frequently observed postviral chronic fatigue and affective features after coronavirus disease (COVID) are related to high levels of inflammation, oxidative damage, and lowered antioxidant defenses [90]. Depressive symptoms in COVID-19 survivors were also associated with high levels of inflammation, that correlated with low levels of glutathione in the anterior cingulate cortex [91]. Vitamin E, an antioxidant compound, has also been reported to be beneficial for the oxidative stress and inflammation of depressive patients [92].

All this evidence supports the connection between the inflammatory processes present in depression and oxidative stress. Altogether, these mechanisms promote the development and onset of depression, being important therapeutic targets. Table 2 summarizes data about the in vivo studies presented in this subsection.

### 3.3. Oxidative Stress and Depression’s Associated Serotonin Imbalance

**Scheme 1:**
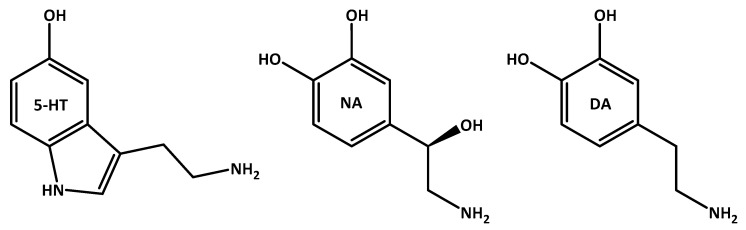
Neurotransmitters of the brain: serotonin (5-HT), noradrenaline (NA)/norepinephrine, and dopamine (DA). 5-HT and NA strongly influence mental behavior patterns, while dopamine is involved in movement.

One of the possible mechanisms that are described to lead to the development of depression is based on the monoamine hypothesis. Indeed, this hypothesis describes that altered levels of the monoamine neurotransmitters serotonin (5-HT), noradrenaline (NA), and dopamine (DA) are associated with depression [93] (Scheme 1).

Indeed, 5-HT is particularly described as an important player in the pathogenesis of depression. Some of the most prescribed antidepressants are the selective serotonin reuptake inhibitors (SSRIs). These antidepressants act by inhibiting the reuptake of serotonin, increasing serotonin activity [94]. Thus, the efficacy of SSRIs demonstrates that 5-HT may be highly implicated in depression. However, the association between depression and 5-HT is not totally clear. A recent systematic umbrella review reported no consistent evidence of a clear association between 5-HT and depression [95]. Nevertheless, hundreds of scientific studies focus on the role of 5-HT and 5-HT-related pathways in depression, being an important topic of studies on the long way to fully understand how depression works.

Oxidative stress leads to a high number of neurotoxic substances by the oxidation of the 5-HT precursor tryptophan. Oxidation-specific epitopes such as MDA are molecules known to have extremely proinflammatory properties, being correlated to depression by interacting with pathways such as tryptophan/kynurenine. Indeed, tryptophan may be metabolized to kynurenine through proinflammatory cytokines and directly by ROS. This molecule may be further metabolized to prooxidant compounds, 3-hydroxykynurenine and quinolinic acid, associated with the pathogenesis of depression [71,96].

Several research works demonstrate the connection between oxidative stress and serotonin in the context of depression. A recent study demonstrated that selenium-modified fluoxetine derivatives simultaneously target 5-HT reuptake (antidepressant action) and oxidative stress, leading to promissory results [97]. Additionally, another study revealed that mirtazapine and L-tryptophan (both players in serotonergic-related pathways) counteracted the cellular stress induced by hydrogen peroxide, highlighting the role of serotonergic pathways in oxidative stress [98]. Another study revealed that saffron intake also protected human neurons from oxidative stress, stimulating the production of dopamine, 5-HT, and BDNF. In addition, saffron inhibited the expression of the 5-HT transporter (SERT). Indeed, all these data provided new insights into the context of oxidative stress related to depression [99]. Other data also revealed that oxidative DNA and RNA damage was attenuated after the treatment with SSRIs, in the case of unipolar depression [100]. Nacre extract from pearl oyster also suppressed LPS-induced depression and anxiety in mice. Indeed, the high levels of oxidative stress induced by LPS were accompanied by changes in 5-HT receptors 5-HT1A and 5-HT2A, and BDNF, revealing an antidepressant action [101]. Persimmon leaf extract is also associated with antioxidant properties. The administration of this extract to depressed mice prevented dendritic spine loss through the inhibition of 5-HT reuptake, increasing 5-HT brain levels, alleviating the depressive-like behavior of the animals [102]. Another study supported the connection between 5-HT and oxidative stress. This study in murine RAW264.7 macrophages revealed that 5-HT and its metabolites reduced oxidative stress and prevented the production of inflammatory cytokines by macrophages [103]. In a postpartum depression rat model, supplementation with the probiotic Lactobacillus casei improved depression-like behaviors. Indeed, this probiotic reversed rats’ gut microbiota, leading to several processes such as enhanced expression of monoamines and the BDNF/ERK1/2 pathway, and decreased levels of oxidative stress (suppressed the increase in MDA and promoted SOD activity) [104].

Altogether, these studies highlight the connection between the 5-HT imbalance present in depression and oxidative stress. Table 3 summarizes data about the in vivo studies presented in this subsection.

### 3.4. Oxidative Stress and Depression’s Associated Synaptic Plasticity and Neurogenesis Imbalance

Synaptic plasticity is a process that refers to the alteration of the strength or efficacy of synaptic transmission at existing synapses. Impairments in this mechanism contribute to several neuropsychiatric disorders such as depression [105]. BDNF is a neurotrophin involved in synaptic plasticity, extensively studied in the context of depression. Dysfunctions of this neurotrophin culminates in imbalances of synaptic plasticity and decreased excitatory neurons and glutamate, promoting depression [106]. Indeed, levels of BDNF mRNA were reduced in postmortem samples collected from depressed brains (versus control) [107] and as a result of suicide [108]. Additionally, increased BDNF expression was found in the hippocampus in patients treated with antidepressants, compared with antidepressant-untreated patients [109]. BDNF also plays a crucial role in hippocampal neurogenesis. Neurogenesis is the process of the formation of neurons de novo. In adults, it is known to occur in the lateral subventricular zone and in the dentate gyrus of the hippocampus. Several factors may affect this process. Indeed, in animal models, enhanced neurogenesis after regular physical exercise has been reported, whereas stress (acute or chronic) is known to decrease this process [110,111,112]. Additionally, corticosteroids reduce neurogenesis and BDNF, other trophic factors (such as epidermal growth factor (EGF)), and 5-HT are known to enhance this process [110]. Depression is also connected with impairment of hippocampal neurogenesis. Indeed, studies report low levels of typical biomarkers of neurogenesis, such as reduced volume of the dentate gyrus and reduced vascularization of the neurogenic niche [113,114,115]. Additionally, after SSRI treatment, an increase in neurogenic hippocampal markers was observed in depressed individuals, such as neural progenitor cells in the dentate gyrus [113,116]. Moreover, there are other situations where there is an association between depression and impairment of neurogenesis, for instance, it is known that Western diets are associated with a reduction in the neurogenic process [117] and with an increased risk of depressive symptoms in adolescents [118].

Studies support the link between oxidative stress and neurogenesis and synaptic plasticity impairments in depression, mainly by connecting them with BDNF. A recent study highlighted that the low levels of neurogenesis present in stress-induced depressed mice were rescued by upregulation of the mitochondrial antioxidant sirtuin 3, being a promising strategy to confer stress resilience and improve depressed behavior [119]. The administration of tilapia skin peptides to mice also improved depression-like behavior by regulating oxidative stress and neurogenesis. Indeed, the data obtained suggest that the BDNF/TRKB/CREB pathway may be involved in the antidepressant effects of these peptides, ameliorating neurogenesis and neural apoptosis. Additionally, this compound enhanced the Nrf2/HO-1 pathway, that regulates the expression of numerous antioxidant genes [120]. The signaling pathway brain-derived neurotrophic factor (BDNF)-protein kinase B (Akt)/ signal-related kinase 2 (ERK2) was also restored after administration of flower essential oil of Tagetes minuta. In this study, oxidative stress was also mitigated, attenuating depressive-like behavior in mice [87]. Another study revealed that melatonin increased antioxidant markers and increased neurogenesis in the hippocampus and prefrontal cortex of rats previously treated with methotrexate. In fact, melatonin ameliorated the antioxidant defenses of the animals by improving the Nrf2 and BNDF expression. It also upregulated synaptic plasticity and enhanced the expression of doublecortin, an important marker of neurogenesis [121]. The administration of the antioxidant carvedilol to depressed mice also increased brain glutathione and BDNF concentrations, and decreased MDA levels, presenting antidepressant-like effects [122]. Similar results were observed with the administration of luteolin-7-O-glucuronide to mice. Indeed, this compound has reported antioxidant properties and improved depression-like behavior, activating BDNF signaling and, thus, modulating neurogenesis and neuroplasticity [123]. Rosmaniric acid also reversed LPS-induced depressive behaviors in mice by promoting the expression of the BDNF/Nrf2 pathway, also leading to the expression of antioxidant enzymes such as heme oxydase-1 and NAD(P)H quinone dehydrogenase 1 (NQO1), downregulating the expression of proinflammatory genes [124]. Both celastrol and thymoquinone also alleviated depressive and anxiety behaviors in rats by reverting the concentration of acetylcholine, dopamine, and serotonin, previously decreased by exposure to aluminum chloride. Additionally, these compounds increased BDNF expression and downregulated the oxido-inflammatory markers (such as MDA and IL-6) in the brains of the rats [125]. In SH-SY5Y cells, walnut polyphenols and the active compound urolithin A also improved oxidative damage caused by hydrogen peroxide, enhancing PKA/CREB/BDNF signaling and promoting neuroprotection [126].

All this evidence serves as support for a connection between the imbalance in ROS production (oxidative stress) and neurogenesis and synaptic plasticity impairment, being important in the pathogenesis of depression. Table 4 summarizes data about the in vivo studies presented in this subsection.

## 4. Conclusions

Depression is a prevalent, highly debilitating, and complex disease. Understanding the molecular pathways present in this disease could lead to new therapeutic options, highly benefiting the quality of life of patients. Indeed, oxidative stress is a major player in the pathogenies of depression, being connected with the imbalances in the stress response, neuroinflammation, serotonin, neurogenesis, and synaptic plasticity that are present in this disease, promoting its onset and development. Thus, counteracting the harmful effects of high levels of oxidative stress is a promissory strategy for the treatment of depression.

The detailed study of the connection of oxidative stress with pathophysiological mechanisms such as the stress response, neurogenesis, synaptic plasticity imbalances, and other less studied processes such as hypoxia in depression is important to lead to a new era of treatment and understanding of this disease. All the presented data make us realize that exploring all these mechanisms allows us to find an answer on the much-needed effective therapy of this disease. In fact, future efforts are needed to keep investigating the molecular/cellular details involved in the pathogenesis of depression, also aiming at an effective personalized therapy, the future of medicine.

## Figures and Tables

**Table 1:** Compound doses, route of administration, strains used, and main findings of the in vivo studies presented in this subsection.

Compound	Strain	Doses	Route of Administration	Main Findings
Cholecalciferol [57]	Male Swiss mice	2.5, 7.5, 25 μg/kg	Oral	Counteracted depressive-like behavior and oxidative stress
Lutein [58]	Male Swiss mice	0.1, 1, and 10 mg/kg	Oral	Counteracted depressive-like behavior and oxidative stress
Crocin-I [59]	C57BL/6 J mice	20 and 40 mg/kg	Oral	Counteracted neuroinflammation and oxidative damage
Catalpol [60]	Kunming mice	20 mg/kg	Intragastric	Inhibited the HPA axis hyperactivity, central inflammation, and oxidative damage
Myrcia pubipetala Miq. [61]	Male Swiss mice	50, 100, or 150 mg/Kg	Oral	Antioxidant effects
L-Cysteine [63]	Male albino rats	150 mg/kg/mL	Oral	Reduced corticosterone levels and increased antioxidant defenses

**Table 2:** Compound doses, route of administration, strains used, and main findings of the in vivo studies presented in this Section 3.2.

Compound	Strain	Doses	Route of Administration	Main Findings
Muscone [72]	C57BL/6 mice	10 mg/kg	Intragastric	Ameliorated depression-like behavior by regulating inflammatory and oxidative stress markers
Apple phenolic extracts [73]	Kunming mice	200 ppm	Intragastric	Regulation of oxidative stress, neuroinflammation, and apoptosis
Cinnamic acid [74]	C57BL/6J mice	50, 100 and 200 mg/kg	Intragastric	Improved depressive-like behavior by inhibiting neuroinflammation and oxidative stress
Curcumin [77]	Wistar rats	50 mg/kg	Intragastric	Attenuated anxiety/depression-like behaviors, decreasing oxidative stress and neuroinflammation
p-Coumaric acid [78,79]	Sprague Dawley rats [78]; male Institute of Cancer Research (ICR) mice [79]	25, 50, and 100 mg/kg [78]; 75 mg/kg [79]	Intraperitoneal injection [78,79]	Protective role against inflammation and oxidative stress [78,79]
Quercetin [80]	Zebrafish	50 and 100 mg/kg	Intraperitoneal injection	Alleviated oxidative stress and neuroinflammation
LQFM212 [84]	Albino Swiss mice	54 μmol/kg (18.8 mg/kg)	Oral	Antioxidant effects and ameliorated behavioral, inflammatory, and oxidative changes
Resveratrol [86]	Wistar rats	20, 40, or 80 mg/kg	Intraperitoneal injection	Antioxidant, anti-inflammatory, and antidepressant effects
Flower essential oil of Tagetes minuta [87]	Adult *Swiss* mice	10 and 50 mg/kg	Intragastric	Attenuated depressive-like behavior by reducing oxidative stress and inflammation and control of BDNF-related pathways
Astilbin [89]	C57/BL6 mice	2, 4, 6, and 8 mg/kg	Intraperitoneal injection	Ameliorated depressive-like behavior by regulating astrocyte-mediated neuroinflammation

**Table 3:** Compound doses, route of administration, strains used, and main findings of the in vivo studies presented in this subsection. CFU, colony-forming unit.

Compound	Strain	Doses	Route of Administration	Main Findings
Saffron extract [99]	Healthy volunteers	300 mg	Oral	Protected human neurons from oxidative stress, stimulating the production of dopamine, 5-HT, and BDNF
Escitalopram [100]	Patients with unipolar depression	10–20 mg	Oral	Reduced DNA and RNA damage from oxidation
Duloxetine [100]	Patients with unipolar depression	30–120 mg	Oral	Reduced DNA and RNA damage from oxidation
Nacre extract [101]	ICR mice	50 and 100 mg/kg	Intraperitoneal injection	Suppressed depression and anxiety behavior, attenuating the high levels of oxidative stress
Persimmon leaf extract [102]	Single-housed CD-1 mice and C57BL/6 mice	30.0–60.0 mg/kg	Intragastric	Prevented dendritic spine loss, alleviating the depressive-like behavior
Lactobacillus casei [104]	Sprague Dawley rats	8 × 10^8^ CFU/kg/day	Intragastric	Improved depression-like behaviors, increased expression of monoamines, and decreased levels of oxidative stress

**Table 4:** Compound doses, route of administration, strains used, and main findings of the in vivo studies presented in this Section 3.4.

Compound	Strain	Doses	Route of Administration	Main Findings
Tilapia skin peptides [120]	C57BL/6 mice	250, 500, and 1000 mg/kg	Intraperitoneal injection	Improved depression-like behavior by regulating oxidative stress and neurogenesis
Flower essential oil of Tagetes minuta [87]	Adult Swiss mice	10 and 50 mg/kg	Intragastric	Attenuated depressive-like behavior by reducing oxidative stress and control of BDNF-related pathways
Melatonin [121]	Sprague Dawley rats	8 mg/kg	Intraperitoneal injection	Increased antioxidant markers and increased neurogenesis
Carvedilol [122]	Adult Swiss mice	5 and 10 mg/kg	Oral	Increased glutathione and BDNF concentrations, and decreased oxidative stress, presenting antidepressant-like effects
Luteolin-7-O-glucuronide [123]	C57BL/6 mice	0.3, 1, and 3 mg/kg	Oral	Antioxidant properties and improved depression-like behavior, activating BDNF signaling
Rosmarinic acid [124]	Specific pathogen-free (SPF) C57BL/6 mice	80 mg/kg	Intraperitoneal injection	Reversed depressive behaviors by promoting the expression of BDNF, increased expression of antioxidant enzymes, reduced inflammation
Celastrol [125]	Albino Wistar rats	1 mg/kg	Intraperitoneal injection	Alleviated depressive and anxiety behaviors, increased BDNF expression, and downregulated the oxido-inflammatory markers
Thymoquinone [125]	Albino Wistar rats	10 mg/kg	Intraperitoneal injection	Alleviated depressive and anxiety behaviors, increased BDNF expression, and downregulated the oxido-inflammatory markers
